# Radiomics Signature of Epicardial Adipose Tissue for Predicting Postoperative Atrial Fibrillation after Off-Pump Coronary Artery Bypass Surgery

**DOI:** 10.31083/j.rcm2411327

**Published:** 2023-11-23

**Authors:** Yisen Deng, Zhan Liu, Xuming Wang, Xixi Gao, Zhaohua Zhang, Dingkai Zhang, Mingyuan Xu, Haijie Chen, Xueqiang Fan, Yuguang Yang, Zhidong Ye, Peng Liu, Jianyan Wen

**Affiliations:** ^1^Department of Cardiovascular Surgery, Peking University China-Japan Friendship School of Clinical Medicine, 100191 Beijing, China; ^2^Department of Cardiovascular Surgery, China-Japan Friendship Hospital, 100029 Beijing, China

**Keywords:** atrial fibrillation, radiomics, coronary artery bypass surgery, epicardial adipose tissue, non-enhanced CT

## Abstract

**Background:**

Postoperative new atrial fibrillation (POAF) is a commonly observed complication after off-pump coronary artery bypass surgery (OPCABG), and models based on radiomics features of epicardial adipose tissue (EAT) on non-enhanced computer tomography (CT) to predict the occurrence of POAF after OPCABG remains unclear. This study aims to establish and validate models based on radiomics signature to predict POAF after OPCABG.

**Methods:**

Clinical characteristics, radiomics signature and features of non-enhanced CT images of 96 patients who underwent OPCABG were collected. The participants were divided into a training and a validation cohort randomly, with a ratio of 7:3. Clinical characteristics and EAT CT features with statistical significance in the multivariate logistic regression analysis were utilized to build the clinical model. The least absolute shrinkage and selection operator (LASSO) algorithm was used to identify significant radiomics features to establish the radiomics model. The combined model was constructed by integrating the clinical and radiomics models.

**Results:**

The area under the curve (AUC) of the clinical model in the training and validation cohorts were 0.761 (95% CI: 0.634–0.888) and 0.797 (95% CI: 0.587–1.000), respectively. The radiomics model showed better discrimination ability than the clinical model, with AUC of 0.884 (95% CI: 0.806–0.961) and 0.891 (95% CI: 0.772–1.000) respectively for the training and the validation cohort. The combined model performed best and exhibited the best predictive ability among the three models, with AUC of 0.922 (95% CI: 0.853–0.990) in the training cohort and 0.913 (95% CI: 0.798–1.000) in the validation cohort. The calibration curve demonstrated strong concordance between the predicted and actual observations in both cohorts. Furthermore, the Hosmer-Lemeshow test yielded *p* value of 0.241 and 0.277 for the training and validation cohorts, respectively, indicating satisfactory calibration.

**Conclusions:**

The superior performance of the combined model suggests that integrating of clinical characteristics, radiomics signature and features on non-enhanced CT images of EAT may enhance the accuracy of predicting POAF after OPCABG.

## 1. Introduction

Coronary artery disease (CAD) has become a global health problem, and coronary artery bypass grafting (CABG) remains an important treatment [1]. Postoperative new atrial fibrillation (POAF) is a commonly observed complication after CABG, and the incidence ranges between 28%–33% [2]. Although off-pump coronary artery bypass surgery (OPCABG) has shown potential in reducing complications by avoiding cardiac intubation and minimizing the release of cytokines and inflammatory mediators associated with cardiopulmonary bypass, its impact on POAF rates has not demonstrated a significant decrease [3]. POAF may lead to prolonged use of ventilator, decreased blood pressure, heart failure, myocardial ischemia, and stroke, resulting in multiple complications, including an increased risk of short-term and long-term mortality [4, 5]. It is therefore critical to understand the occurrence of POAF after OPCABG and screen risk factors in order to prevent POAF.

Epicardial adipose tissue (EAT) is a unique adipose depot that gets its blood supply from small branches of the coronary artery and is directly adjacent to coronary arteries and myocardium [6]. EAT refers to the adipose tissue found between the surface of the myocardium and the visceral pericardium, which is mostly located in the atrioventricular sulcus and interventricular sulcus, but can also be observed on the surface of the coronary artery or even inside the myocardial tissue. Previous studies have revealed that the epicardial adipose volume measured by computer tomography (CT) is an independent risk factor for atrial fibrillation (AF) [7]. Several studies have reported an association between EAT and the incidence, severity, and recurrence of atrial fibrillation [8, 9]. In addition, the increase in fat thickness near the left atrium was found to be significantly correlated with atrial fibrillation burden [7]. Yet the mechanism behind atrial fibrillation caused by pathological changes of epicardial adipose tissue remains unknown.

Recently, radiomics has attracted extensive attention for its ability to extract high-throughput data from medical images. Machine learning and other methods can then be used to evaluate the features of the images to find novel applications [10, 11]. Models based on radiomic signatures can provide guidance for doctors and improve the accuracy of diagnosis and prognosis. It has been demonstrated that radiomics has a unique value in the identification of coronary artery plaques and in discriminating between hypertensive heart disease and hypertrophic cardiomyopathy [12, 13]. Yang* et al*. [8] reported that radiomics signatures of EAT around the left atrium have a promising value in differentiating atrial fibrillation subtypes and predicting the recurrence of atrial fibrillation. On the basis of these previous findings, we suggest that radiomics features of EAT may provide accurate prediction of POAF.

Coronary artery computer tomography angiography (CCTA) is extensively employed for the diagnosis of CAD, but some patients received coronary angiography to diagnose CAD rather than CCTA, so as to perform percutaneous transluminal coronary intervention (PCI) at the same time if necessary [14]. Moreover, iodine contrast agents can increase the attenuation of fat around the coronary artery in inflammatory conditions, and non-contrast CT images might reflect more reliably radiological features [15]. In our institution, non-contrast CT scans were performed commonly as part of preoperative evaluation for CABG. Thus, we aims to establish and validate models based on radiomics features of EAT on non-enhanced CT images to predict the occurrence of POAF after OPCABG, which might contribute to the identification of high-risk individuals and improve the prognosis of patients through active intervention.

## 2. Materials and Methods

### 2.1 Patient Selection

The ethics board of the China-Japan Friendship Hospital granted approval for this retrospective study, and informed consent was waived accordingly. Totally, 96 patients who underwent OPCABG between September 2017 and May 2022 were included. The patients were randomly allocated into a training cohort (n = 67) and a validation cohort (n = 29) at a ratio of 7:3.

### 2.2 Clinical Features

We retrieved preoperative demographics data, electrocardiogram, hematologic examination, and echocardiography from the medical information system. All patients underwent continuous electrocardiographic monitoring during the postoperative period, every identified arrhythmia event was then confirmed by a cardiologist. Continuous electrocardiographic monitoring was performed on all patients during the postoperative period, and any identified arrhythmia event was subsequently verified by a cardiologist. The criteria used to define POAF were any recorded AF episode that lasted for more than 30 seconds, documented either by continuous telemetry throughout the patient’s hospital stay or by a 12-lead electrocardiogram conducted on a daily basis [16].

### 2.3 Computer Tomography Scan

All the patients received a CT examination within 7 days before OPCABG. CT scans were performed by a multi-detector CT system (GE Revolution CT/256, GE Healthcare, Milwaukee, WI, USA), using scanning parameters of low dose CT in chest: tube ball voltage 120 kV, current 300 mAs, slice thickness 5 mm. The scanning region ranged from the tip of the lung to the lower edge of the second lumbar vertebra (L2). The patients were instructed to lie on their backs, raise their hands, and hold their breath for a single scan at the end of inspiration.

### 2.4 EAT Segmentation 

We performed EAT segmentation for radiomics analysis through the three dimensions (3D) slicer software (version 4.13.0, Harvard, Boston, MA, USA). Two experienced radiologists independently delineated the volumes of interest (VOIs) along the borders of the fibrous pericardium on cardiac axial slices, from the bifurcation of the pulmonary trunk to the lowest slice of pericardium. Radiologists were blinded to the patients’ clinical features. EAT was identified using a segmentation algorithm that applied a densitometric threshold (density range between –190 HU (Hounsfield unit) and –30 HU). Once the delineation was completed, the 3D slicer software automatically calculated the EAT volume and radiodensity. One month after the initial delineation, another reader repeated the process of outlining the regions of interest (ROIs) in all patients. The mean values of EAT volume and radiodensity were recorded based on three separate measurements.

### 2.5 Feature Extraction

The VOI image normalization and resampling were performed using the Pyradiomics package of Python Software (version 3.7, Python Software Foundation, DE, USA) as mentioned in our prior study [17]. In order to address the curse of dimensionality, which is particularly evident in high-dimensional data, we employed feature extraction techniques such as laplacian of gaussian (LoG) filters (sigma value of 1.0, 2.0, 3.0, 4.0, and 5.0) and wavelet transformation [18]. These techniques allowed us to mitigate the issue by reducing unnecessary features and extracting more relevant and informative features for our radiomics model. We subsequently extracted 1218 quantitative radiomics features from the VOI in original image and from its corresponding filtered image, including shape features (14), first order statistics (18), gray-level co-occurrence matrix features (22), gray-level run-length matrix features (16), gray-level size-zone matrix features (16), gray-level dependence matrix features (14), wavelet features (688), and Laplacian of Gaussian filters features (430).

### 2.6 Feature Selection

We conducted feature selection in the training cohort. The radiomics features extracted from the training cohort were normalized to eliminate differences caused by varying value scales. The features of the validation cohort were standardized using the mean and standard deviation values calculated from the training cohort. The reproducibility of the radiomics features was evaluated through both intra-class and inter-class correlation coefficients (ICC). Features with an ICC >0.9 were regarded as reproducible and were selected for further analysis. We calculated the Spearman or Pearson correlation coefficients for each pair of features, and excluded those with a correlation coefficient >0.9. We then employed the least absolute shrinkage and selection operator (LASSO) algorithm to identify relevant radiomics features that had non-zero coefficients.

### 2.7 Development and Validation of Prediction Model

Univariate logistic regression analyses were performed to analyze the clinical characteristics and EAT CT features, including EAT volume and radiodensity, in the training cohort. The features with statistical significance in the univariate analysis will be further analyzed using multivariate logistic analysis. Features with statistical significance in the multivariate analysis were utilized to build clinical model. Selected radiomics features with non-zero coefficients were used to develop a radiomics signature through a linear regression model, where each feature was weighted by its respective coefficient. The combined model was established by integrating the radiomics signature, the clinical characteristics and features of CT images. A nomogram was generated to visualize the combined model.

### 2.8 Model Performance Assessment

**Fig. 1.**
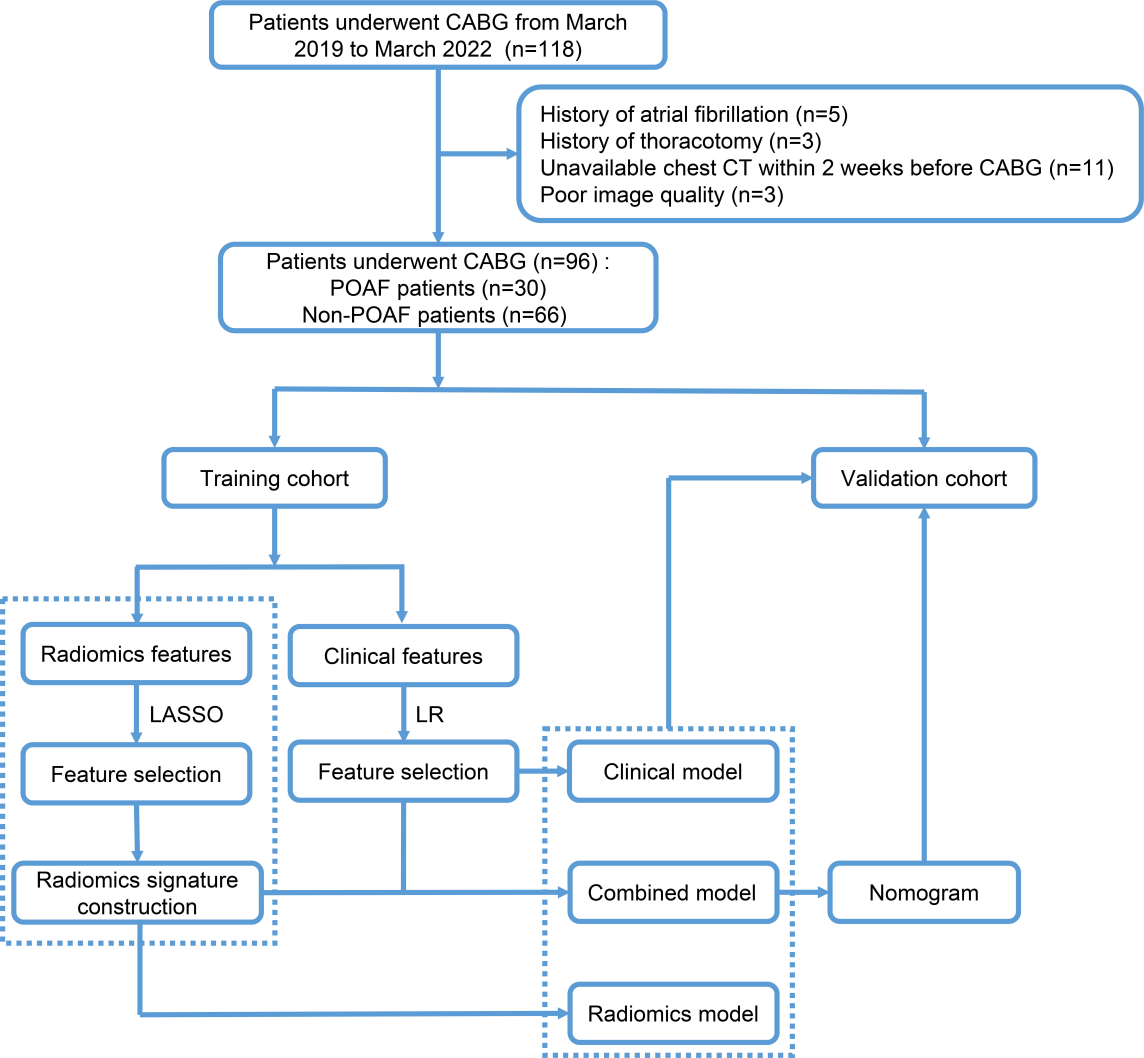
**Flow diagrams of patient selection and study design**. CABG, coronary artery bypass surgery; CT, computer tomography; POAF, postoperative new atrial fibrillation; LASSO, the least absolute shrinkage and selection operator; LR, logistic regression.

**Fig. 2.**
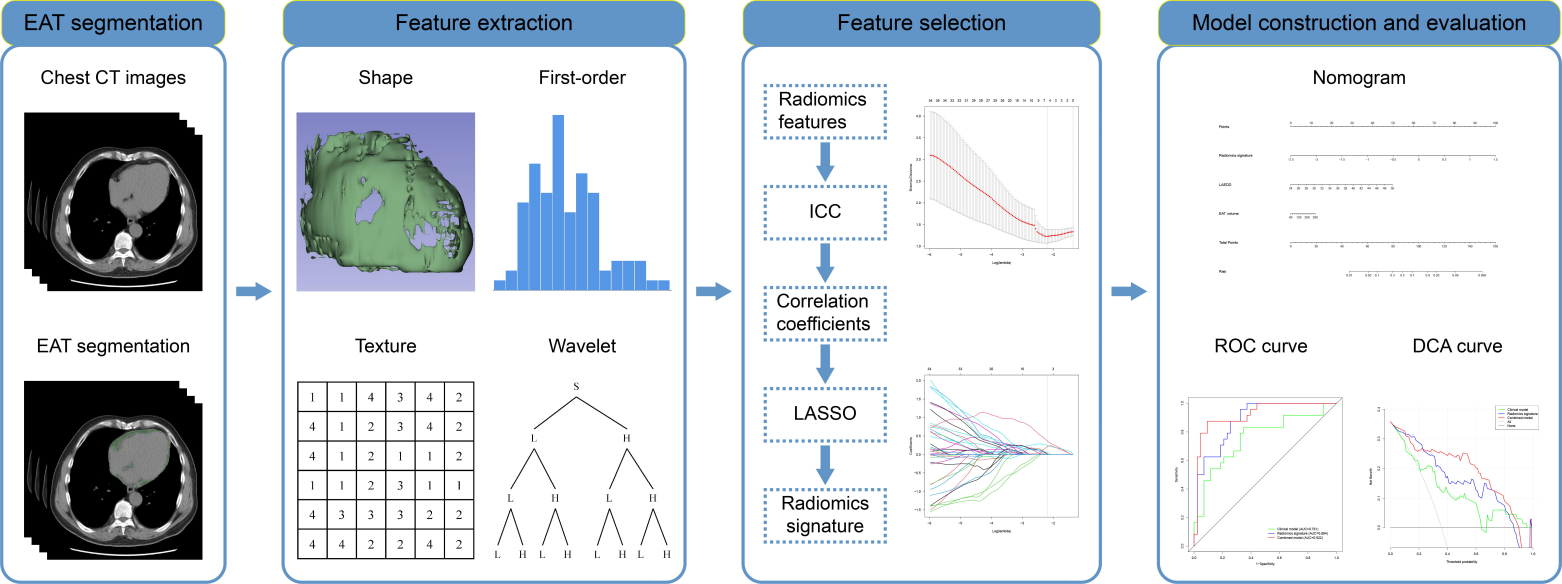
**Flowchart of specific radiomics signature analysis**. EAT, epicardial adipose tissue; CT, computer tomography; ICC, inter-class correlation coefficients; LASSO, the least absolute shrinkage and selection operator; ROC, receiver operating characteristic; DCA, decision curve analysis.

The performance of the models was evaluated using the area under curve (AUC) in the receiver operating characteristic (ROC) curves. The AUCs of the three models were compared through DeLong’s test. Calibration of the prediction model was evaluated by calibration curves and Hosmer-Lemeshow test. Bootstrap validation with 1000 resamples was performed to assess the accuracy of the calibration curve and ideal curve overfit. Furthermore, we conducted decision curve analysis (DCA) to evaluate the clinical utility of the prediction model through calculating the net benefit at various threshold probabilities. The three models were subsequently validated in the validation cohort. The flow diagrams of this study and the process of specific radiomics signature analysis are shown in Figs. 1,2, respectively.

### 2.9 Statistics Analysis

In this study, statistical analyses were performed using R software (version 3.5.1, R Foundation for Statistical Computing, Vienna, Austria) and SPSS (version 26.0, IBM Corp., Armonk, NY, USA). Continuous variables were expressed as mean ± standard deviation (SD), while categorical variables were presented as numbers (percentage). The clinical characteristics and features of CT images in training and validation cohorts were compared with *t*-test (for continuous variables) and chi-square test (for categorical variables). Probability values were 2-sided, and *p* < 0.05 was considered statistically significant.

## 3. Results

### 3.1 Clinical Characteristics

This study retrospectively included a total of 96 patients with CAD, among whom 30 patients had POAF (31.3%) and 66 patients did not have POAF (68.7%). There were 67 patients (POAF: 24, non-POAF: 43) in the training cohort and 29 patients (POAF: 6, non-POAF: 23) in the validation cohort. There were no significant differences in the clinical features between two cohorts (Table 1).

**Table 1.** **Clinical characteristics of patients in the training cohort and validation cohort**.

Characteristics		Training cohort	Validation cohort	*p* value
		(n = 67)	(n = 29)	
Gender, n (%)				0.096
	Male	50 (74.6)	26 (89.7)	
	Female	17 (25.4)	3 (10.3)	
Age (years)		64.13 ± 10.08	64.38 ± 11.83	0.918
BMI (Kg/$m^{2}$)		25.31 ± 3.97	25.11 ± 4.27	0.827
Smoking history, n (%)		28 (41.8)	11 (37.9)	0.724
Alcohol abuse, n (%)		11 (16.4)	7 (24.1)	0.374
High blood pressure, n (%)		53 (79.1)	21 (72.4)	0.474
Diabetes, n (%)		39 (58.2)	15 (51.7)	0.556
Hyperlipidemia, n (%)		37 (55.2)	17 (58.6)	0.758
Acute MI, n (%)		16 (23.9)	9 (31.0)	0.463
Bypass number		1.91 ± 0.45	2.10 ± 0.62	0.090
Operation time (h)		4.40 ± 0.82	4.56 ± 1.16	0.454
LAEDD (mm)		38.54 ± 4.75	39.72 ± 4.71	0.262
LVEF (%)		58.66 ± 12.88	57.70 ± 9.14	0.744
CRP (mg/L)		13.50 ± 29.11	14.14 ± 30.92	0.944
WBC (${\mathrm{10}}^{9}$/L)		6.99 ± 1.92	6.92 ± 1.64	0.871
Neutrophils (${\mathrm{10}}^{9}$/L)		4.54 ± 1.56	4.59 ± 1.69	0.899
Hgb (g/L)		126.18 ± 21.52	128.66 ± 24.04	0.619
CK-MB (U/L)		2.13 ± 2.14	0.82 ± 1.23	0.222
BNP (pg/mL)		379.84 ± 669.05	391.00 ± 533.96	0.961
EAT volume (${\mathrm{cm}}^{3}$)		136.50 ± 50.50	138.51 ± 41.84	0.851
EAT radiodensity (HU)		–74.81 ± 5.74	–73.71 ± 5.49	0.384
POAF, n (%)		24 (35.8)	6 (20.7)	0.142

BMI, body mass index; MI, myocardial infarction; LAEDD, left atrial end-diastolic dimension; LVEF, left ventricular ejection fraction; CRP, C-reactive protein; WBC, white blood cell; Hgb, hemoglobin; CK-MB, creatine kinase-MB; BNP, brain natriuretic peptide; EAT, epicardial adipose tissue; POAF, postoperative new atrial fibrillation; HU, Hounsfield unit.

### 3.2 Feature Selection and Radiomic Signature Construction

**Fig. 3.**
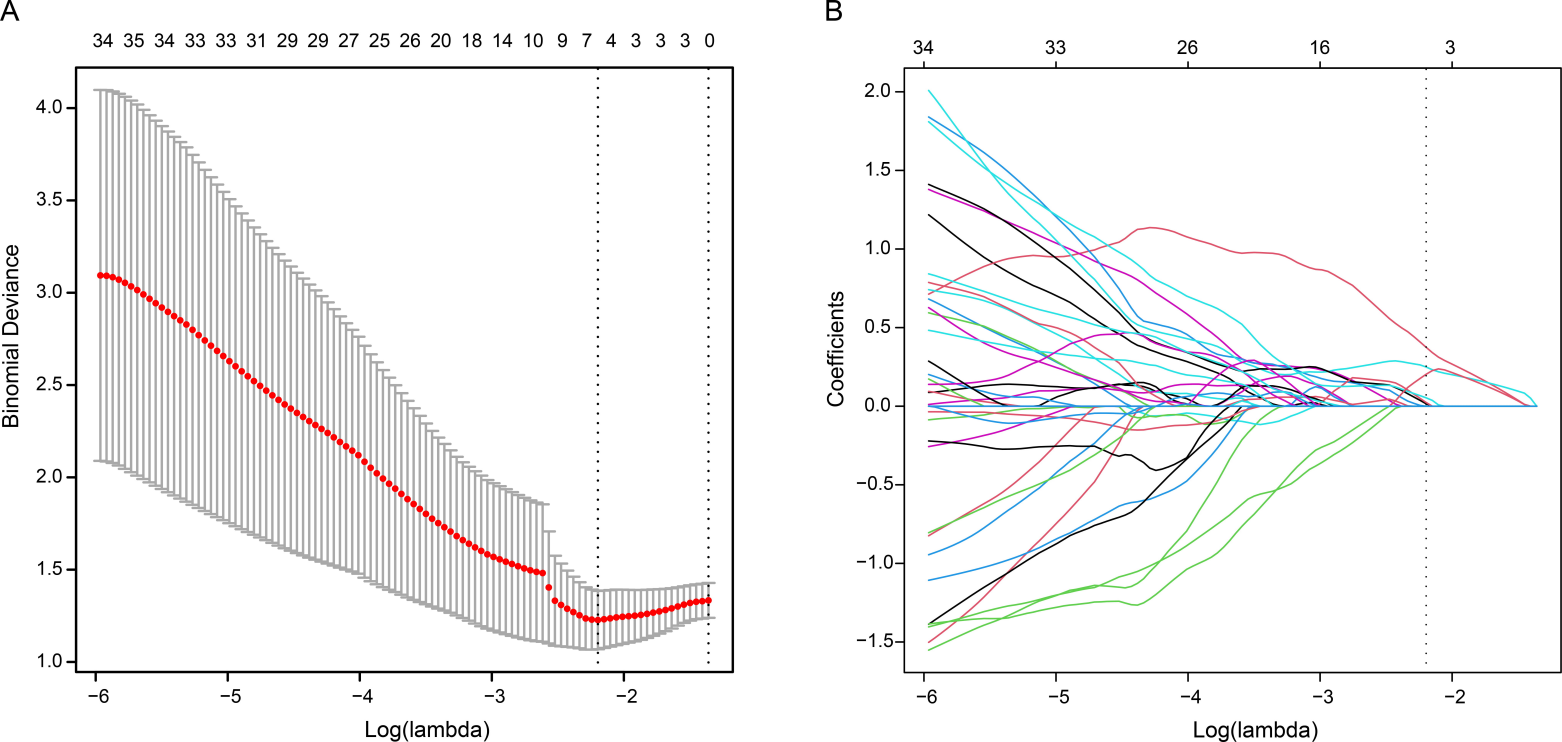
**Identification of significant radiomics features through the least absolute shrinkage and selection operator algorithm (LASSO)**. (A) Coefficient profiles of radiomics features. (B) Six features with non-zero coefficients obtained using optimal lambda λ.

We extracted 1218 radiomics features from each VOI by Pyradiomics. The radiomics features that demonstrated good reproducibility (ICC >0.9) were selected for further analysis. To reduce redundancy, we eliminated features with a high correlation coefficient (≥0.9) as determined by either Spearman or Pearson correlation analysis. The radiomics signature in this study was constructed using six radiomics features, which were selected through the LASSO algorithm. By analyzing the coefficient profiles, we observed the changes in coefficients as the regularization parameter (lambda λ) varied (Fig. 3A). These six features were likely chosen based on an optimal value of the regularization parameter, which aimed to minimize the prediction error or maximize the performance of the model (Fig. 3B). The radiomics signature was calculated as follows:

Radiomics signature = –0.63954826 + 0.37460986 ×original_shape_LeastAxisLength + 0.24917852 × original_shape_Maximum3DDiameter + 0.01537095 × log.sigma.1.0.mm.3D_glcm_Idmn + 0.02363012 × log.sigma.2.0.mm.3D_gldm_DependenceVariance + 0.05315544 × wavelet.LLH_glszm_LargeAreaHighGrayLevelEmphasis + 0.20322590 × wavelet.LLL_glszm_GrayLevelNonUniformity

The radiomics signature was found to be significantly higher in patients with POAF compared to those without POAF in the training cohort (–0.02 ± 0.63 vs –0.98 ± 0.57, *p* < 0.001). Similar trend was observed in the validation cohort (0.06 ± 0.73 vs –0.93 ± 0.56, *p* = 0.001).

### 3.3 Development and Validation of Prediction Models

**Fig. 4.**
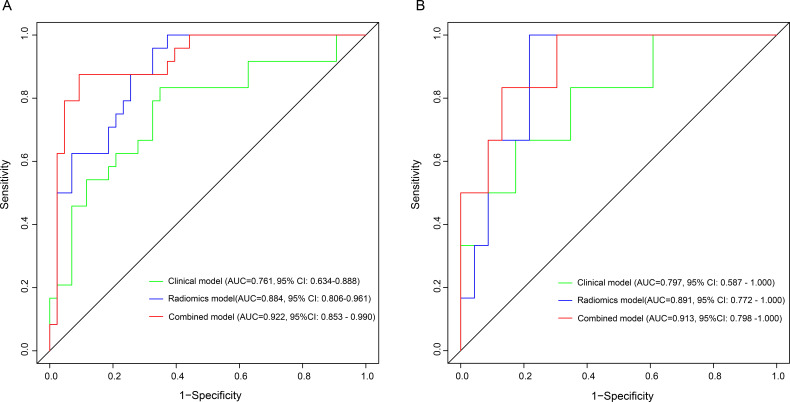
**Comparison of ROCs between clinical model, radiomics model and combined model for predicting AF in the training (A) and validation (B) cohorts**. AUC, the area under the curve; ROC, receiver operating characteristic; AF, atrial fibrillation.

In the training cohort, univariate and multivariate analyses suggested that left atrial end-diastolic dimension (LAEDD) and EAT volume might be independent predictors of POAF (*p* < 0.05, Tables 2,3). We therefore constructed the clinical model using these two independent predictors. The AUC of the clinical model was 0.761 (95% CI: 0.634–0.888) and 0.797 (95% CI: 0.587–1.000) in the training and validation cohort, respectively (Fig. 4). The radiomics model exhibited superior discrimination ability, with an AUC of 0.884 (95% CI: 0.806–0.961) in the training cohort and 0.891 (95% CI: 0.772–1.000) in the validation cohort (Fig. 4).

**Table 2.** **Univariate analysis of clinical characteristics for predicting POAF in training cohort**.

Characteristics		With POAF	Without POAF	*p* value
		(n = 24)	(n = 43)	
Gender, n (%)				0.523
	Male	19 (79.2)	31 (72.1)	
	Female	5 (20.8)	12 (27.9)	
Age (years)		61.63 ± 11.64	65.53 ± 8.93	0.129
BMI (Kg/$m^{2}$)		25.79 ± 4.28	25.05 ± 3.52	0.480
Smoking history, n (%)		11 (45.8)	17 (39.5)	0.616
Alcohol abuse, n (%)		5 (20.8)	6 (14.0)	0.700
High blood pressure, n (%)		17 (70.8)	36 (83.7)	0.213
Diabetes, n (%)		15 (62.5)	24 (55.8)	0.595
Hyperlipidemia, n (%)		13 (54.2)	24 (55.8)	0.897
Acute MI, n (%)		6 (25.0)	10 (23.3)	0.872
Bypass number		1.96 ± 0.46	1.88 ± 0.45	0.521
Operation time (h)		4.43 ± 0.79	4.38 ± 0.85	0.815
LAEDD (mm)		40.54 ± 4.05	37.42 ± 4.78	0.009
LVEF (%)		60.50 ± 13.71	57.62 ± 12.45	0.405
CRP (mg/L)		8.86 ± 9.44	17.61 ± 39.09	0.390
WBC (${\mathrm{10}}^{9}$/L)		6.98 ± 1.82	7.00 ± 2.00	0.977
Neutrophils (${\mathrm{10}}^{9}$/L)		4.42 ± 1.28	4.61 ± 1.70	0.629
Hgb (g/L)		125.25 ± 23.70	126.70 ± 20.49	0.794
CK-MB (U/L)		2.16 ± 2.45	2.11 ± 1.97	0.941
BNP (pg/mL)		96.81 ± 126.30	521.36 ± 782.58	0.102
EAT volume (${\mathrm{cm}}^{3}$)		159.53 ± 61.93	123.64 ± 37.88	0.014
EAT radiodensity (HU)		–73.56 ± 6.24	–75.50 ± 5.38	0.186

BMI, body mass index; MI, myocardial infarction; LAEDD, left atrial end-diastolic dimension; LVEF, left ventricular ejection fraction; CRP, C-reactive protein; WBC, white blood cell; Hgb, hemoglobin; CK-MB, creatine kinase-MB; BNP, brain natriuretic peptide; EAT, epicardial adipose tissue; POAF, postoperative new atrial fibrillation; HU, Hounsfield unit.

**Table 3.** **Multivariate analysis of clinical characteristics for predicting POAF in training cohort**.

Characteristics	OR	95% CI	*p* value
LAEDD	1.183	1.035–1.353	0.014
EAT volume	1.017	1.004–1.030	0.010

LAEDD, left atrial end-diastolic dimension; OR, odds Ratio; CI, credibility interval; EAT, epicardial adipose tissue; POAF, postoperative new atrial fibrillation.

**Fig. 5.**
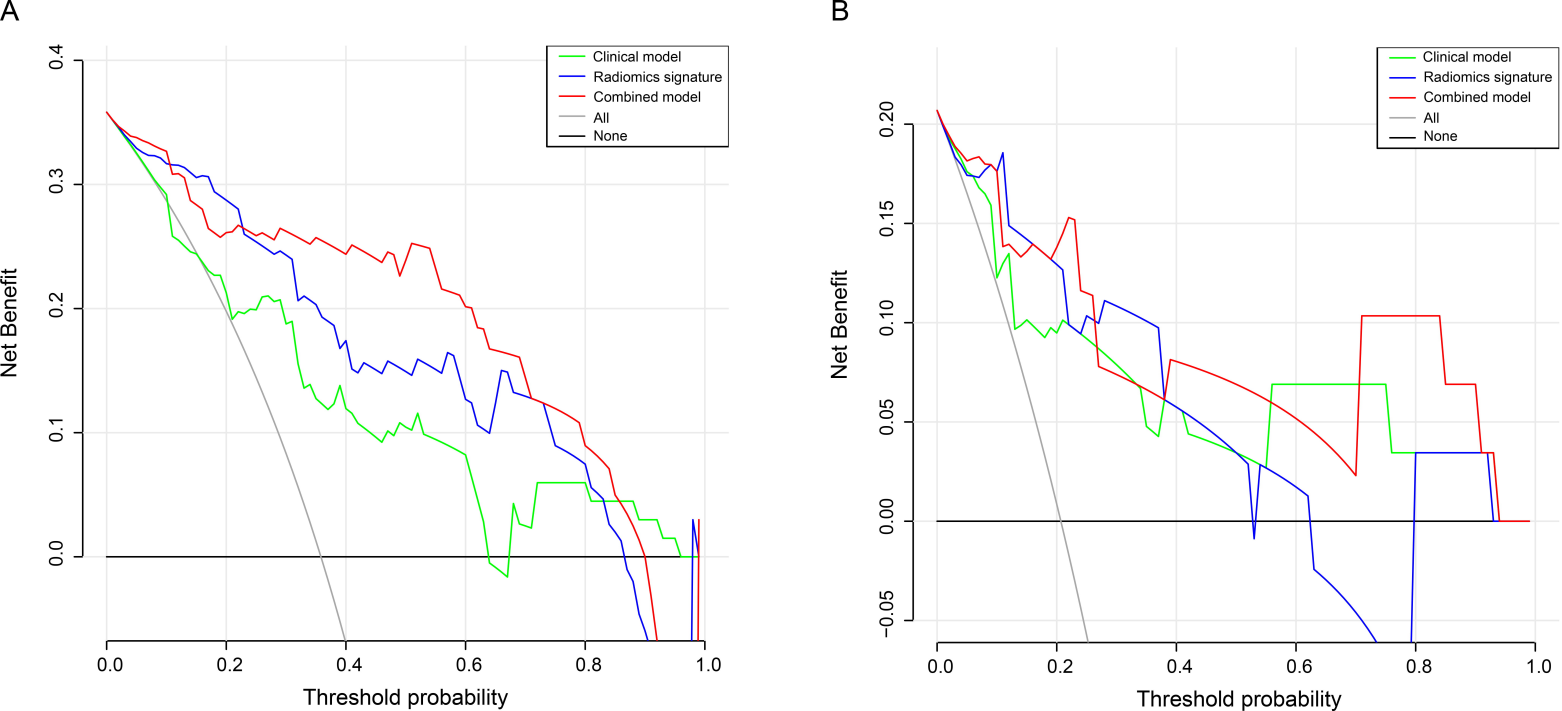
**DCA curves of clinical model, radiomics model and combined model for predicting AF in the training (A) and in the validation (B) cohort**. DCA, decision curve analysis; AF, atrial fibrillation.

**Fig. 6.**
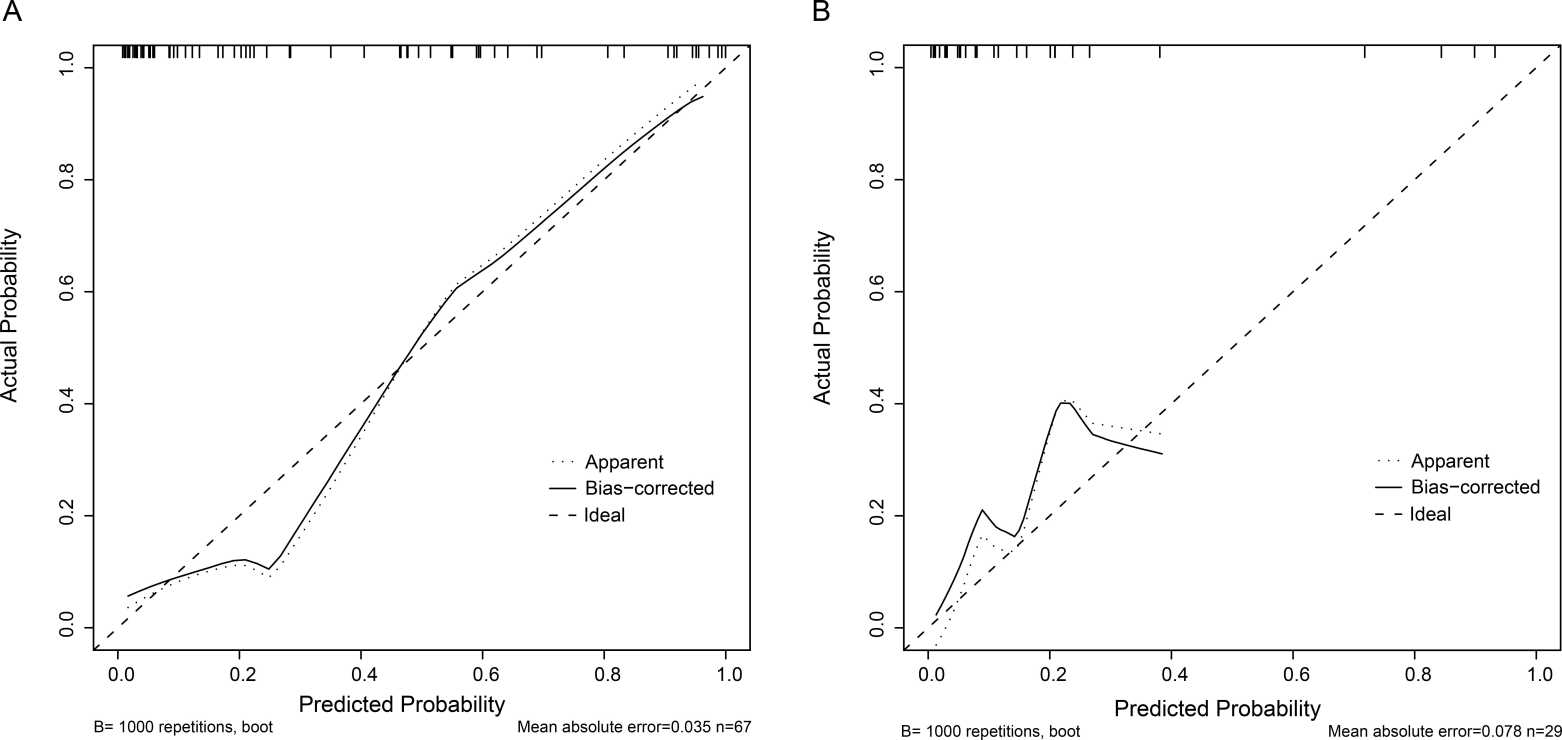
**Calibration curves of the combined model in the training (A) and validation (B) cohorts**. The *p* value of the Hosmer-Lemeshow test is 0.241 in the training cohort and 0.277 in the validation cohort, suggesting a good calibration.

**Fig. 7.**
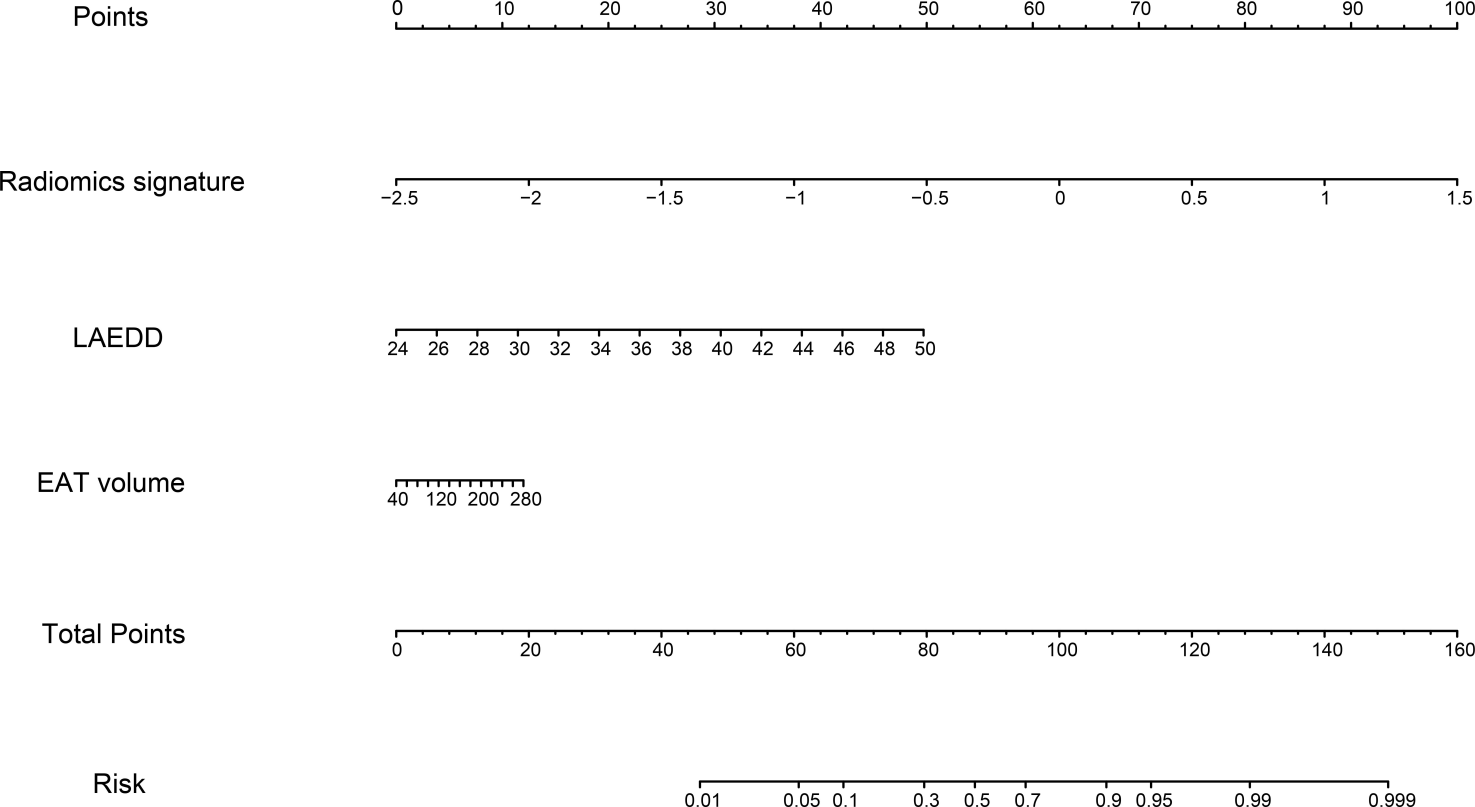
**Nomogram of the combined model based on radiomics signature, LAEDD, and EAT volume**. LAEDD, left atrial end-diastolic dimension; EAT, epicardial adipose tissue.

The combined model was developed using LAEDD, EAT volume, and radiomics signature. The discrimination ability of this model was strong, as evidenced by the AUC of 0.922 (95% CI: 0.853–0.990) in the training cohort and 0.913 (95% CI: 0.798–1.000) in the validation cohort (Fig. 4). The DeLong’s test indicated that the AUCs of the combined model were significantly superior to that of the clinical model in both the training cohort (*p* = 0.003) and validation cohort (*p* = 0.046). The AUCs of the combined model were higher than that of the radiomics model, but the DeLong’s test did not exhibit significant difference in the training cohort (*p* = 0.177) or validation cohort (*p* = 0.530). Nevertheless, the DCA curves showed that in most circumstances using the combined model to identify clinical symptoms would be more clinically beneficial than using the two other separate models (Fig. 5). The calibration curve of the combined model showed good agreement in both the training and validation cohort (Fig. 6). Additionally, the Hosmer-Lemeshow test yielded *p* value of 0.241 in the training cohort and 0.277 in the validation cohort, indicating good calibration of the model. Finally, a nomogram was performed to visualize the combined model (Fig. 7).

## 4. Discussion

Advanced image analysis techniques are used in radiomics to extract high-throughput data from invisible digital images and create datasets that can be mined to reveal associations between different indicators and diseases in order to provide guidance for clinical decisions [19, 20, 21]. Shahzad* et al*. [22] describes a method for automatically measuring the volume of EAT in non-enhanced cardiac CT scans using a multi-atlas segmentation approach. Automated segmentation for measuring EAT offers several advantages, such as reducing the workload on human operators and the associated costs of physicians and technicians. Furthermore, it can provide more precise and consistent results compared to manual segmentation methods [23, 24]. This can aid in early detection, risk stratification, and monitoring of patients, ultimately improving cardiovascular disease management [25]. The ability to predict POAF has implications for risk stratification, implementation of prophylactic measures, perioperative management, tailored treatment strategies, and long-term follow-up. By identifying high-risk patients and intervening appropriately, clinicians can potentially reduce the incidence and severity of POAF, leading to improved patient outcomes and a reduction in associated complications. In this study, we collected radiomics features, clinical characteristics and features of CT images from patients undergoing OPCABG, we then established and evaluated three models for predicting POAF: a clinical model, a radiomics signature model and a combined model. The radiomics signature model, which was based on the radiomics features extracted from CT images, exhibited superior discrimination ability compared to the clinical model. The combined model had the most convincing predictive ability among the three models, suggesting it has great potential in predicting POAF after OPCABG.

Various perioperative conditions may lead to POAF, yet the exact mechanisms behind it remain unknown. Left atrial enlargement is currently recognized as an independent risk factor for POAF. A number of studies have confirmed that patients with left atrial diameter larger than 40mm have a significantly higher risk of POAF [26]. Likewise, we found that LAEDD could act as an independent predictor for POAF. Tsang* et al*. [27] have reported that every 30% increase in left atrial diameter increases the risk of atrial fibrillation by 43% after adjusting for potential confounders such as age, gender, valve diseases and hypertension. The reasons might be that, with the increase of age, cardiac diastolic dysfunctions may occur, which decrease the capability of passive left atrial emptying. The subsequent increase in left atrial filling pressure may lead to progressive enlargement and structural remodeling of the atrium. The changes in physiological characteristics and electrical environment in the left atrium in turns lead to atrial fibrillation [27]. The radiomics signatures capture detailed information about tissue characteristics and spatial patterns within the heart. The interaction between these structural parameters and radiomics signatures may reveal how specific alterations in tissue properties contribute to atrial remodeling, electrical disturbances, and subsequent POAF. Inflammation that leads to structural changes in the left atrium plays a significant role in the occurrence of atrial fibrillation [28, 29], and EAT is one of the sources of inflammatory mediators, which affect the development of CAD [30, 31, 32, 33]. Radiomics signatures may provide insights into localized inflammation within the atrial tissue, highlighting areas of increased inflammatory activity that can further contribute to atrial fibrillation development. Potential atrial fibrillation matrix, intraoperative stimulation and increased secretion of inflammatory factors after operation are all involved in the occurrence of POAF [34]. In addition, inflammatory cells in the atrial tissue have been found in biopsies of patients with AF [8]. Local inflammation may lead to myocardial fibrosis in the atrium, which in turn leads to atrial fibrillation [35]. It was observed that the EAT value in fluorodeoxyglucose positron emission tomography (FDG-PET) images are significantly associated with AF, something that was not observed in subcutaneous adipose tissue [36]. The presence of an inflammatory burden in pericoronary fat is associated with worse outcomes in prediabetic patients undergoing CABG. This inflammatory burden, characterized by altered levels of adipokines and inflammatory markers, contributes to the development and destabilization of atherosclerotic plaques in coronary arteries, thereby increasing the risk of cardiovascular events. In this context, drugs with anti-inflammatory and oxidative effects, such as metformin, play a modulatory role in attenuating the inflammatory burden and improving prognosis [37]. These effects could pass towards the over-expression of inflammatory/oxidative stress molecules, via the sodium-glucose cotransporter 2 (SGLT2)-mediated pathways. The modulation of SGLT2 activity can help mitigate the inflammatory burden, stabilize atherosclerotic plaques, and potentially improve clinical outcomes in prediabetic patients undergoing CABG [38]. By reducing systemic inflammation, modulating adipose tissue inflammation, improving endothelial function, and activating protective pathways, SGLT2 inhibitors offer a multifaceted approach to mitigating inflammation and improving cardiovascular health [39].

We found that EAT volume is an independent predictor for POAF. EAT has been reported to be an independent risk factor for cardiovascular disease and plays a critical role in maintenaning cardiac physiological functions [40]. When EAT increases and infiltrates into the myocardial tissue, myocardial electrical signal conduction is delayed, ultimately promoting the formation of atrial fibrillation matrix [41]. This also leads to dysfunction of cardiomyocytes and promotes myocardial fibrosis, causing structural changes and eventually the occurrence of AF [42]. Higher proportions of fat infiltration were found in patients with POAF, and there was no significant difference in the degree of fat infiltration between left and right atrium [43]. In a study by Yorgun* et al*. [44], the thickness of epicardial adipose tissue (EAT) at various sites in CT scans was measured in 426 patients with atrial fibrillation (AF). The findings revealed a correlation between EAT thickness and AF, with the most significant correlation observed in the left atrium and anterior wall of the right ventricle (r = 0.268, 0.372, *p* < 0.001). It has been observed that the volume model had a significant impact on determining AF [45]. The EAT volume in patients with AF was found to be higher compared to those with normal sinus rhythm (NSR), both in contrast-enhanced CT scans and non-enhanced scans. These findings are in line with the results of a meta-analysis, which demonstrated a higher EAT volume in patients with AF compared to those without AF [46]. In addition to volume, the attenuation of EAT on CT has also gained interest, as the EAT density on CT is partly a reflection of the increased concentration of blood vessels and mitochondria in adipose tissue [47]. Elevated CT attenuation has been identified as a potential indicator of increased cardiac mortality risk and poor prognosis [48]. However, the CT attenuation model derived from contrast-enhanced CT scans did not show significance in determining AF. This could potentially be attributed to the influence of contrast-media enhancement in the EAT, which can interfere with the CT values and make it challenging to differentiate between the AF and NSR groups [45]. We did not observe a significant difference in EAT density between the with POAF group and without POAF group in our study. This finding might be attributed to the limited number of cases included in our study. In general, EAT can exert a proarrhythmogenic effect on the atria through several mechanisms, including the infiltration of adipocytes into the myocardium which contributes to structural remodeling of the left atrium (LA), the release of proinflammatory cytokines that induce inflammation and fibrosis in the myocardium, and the increased adrenergic activation of ganglionic plexuses caused by elevated catecholamine levels or changes in ${\mathrm{Ca}}^{2+}$ currents [49]. The interaction between the identified parameters and significant radiomics signatures may involve a complex interplay of structural, inflammatory, and functional factors, which collectively contribute to the development of POAF. By combining these different aspects, the predictive model captures a broader range of information and provides a more accurate assessment of an individual’s risk for POAF.

A few medical both at the time and after surgery may also lead to the occurrence of postoperative complications, such as myocardial injury and movement of the heart during surgery, or postoperative hypokalemia, which in turn causes increased excitability, decreased conductivity, and increased automaticity of cardiomyocytes. Timely monitoring of blood electrolytes after surgery may make it possible to judge the occurrence of POAF. The incidence of POAF decreased significantly after pericardiectomy of the left atrial posterior wall, which may be related to the complete removal of epicardial adipose tissue in the posterior wall of the left atrium [50, 51]. It was reported that β-blocker and potassium channel blocker amiodarone can significantly reduce the incidence of POAF, while no significant improvements were seen in stroke risk and long-term mortality [52, 53]. The incidence of POAF decreased by 58% after injection of calcium chloride into EAT, but the length of hospitalization of the patients did not improve [54]. Surgical removal of epicardial ganglia can reduce the vagus nerve-related negative frequency or negative conduction to some extent, which provides a theoretical basis for physical intervention in POAF [55]. It was suggested that over-activation of calcium currents and increased levels of sarcoplasmic endoplasmic reticulum calcium ATPase (SERCA) play a significant role in patients who responded to epicardial ablation for persistent atrial fibrillation AF. By targeting SERCA and improving calcium handling, it may be possible to enhance the effectiveness of epicardial ablation and improve outcomes for patients with persistent AF [56].

Radiomics plays a valuable role in monitoring the development and progression of coronary artery atherosclerosis [57]. Studies have shown that the automated system for segmenting the coronary artery, detecting and classifying plaque, and assessing stenosis achieves high levels of accuracy and computational efficiency [25, 58]. Predictive models derived from automated segmentation systems are expected to have a crucial role in future clinical settings. We should recognize that there is a growing demand for the explainability of predictive models in clinical scenarios. While techniques like the LASSO algorithm can effectively identify important features, they may not provide explicit explanations for why those features are relevant. However, there are techniques available that aim to enhance explainability. Techniques such as decision trees, rule-based models, and linear models with explainable coefficients can be explored to make the machine learning model more explainable. Overall, by utilizing machine learning algorithms and incorporating interpretable clinical and radiomic features, it has become possible to gain insights into the models and enable the physician team to provide clinical justifications for the findings [59].

Our study had several limitations. First of all, it is a single center retrospective study with sample size. Although efforts were made to ensure data quality and accuracy, the inherent limitations of retrospective studies, such as potential selection bias, incomplete data, and confounding variables, should be acknowledged. Thus, prospective studies with carefully designed protocols are needed to validate the findings of this study. Secondly, different CT scanners and different image reconstruction algorithms impact the stability of radiomic features. In the future, exploring the correlation between genomic characteristics and radiomics of EAT may reveal a promising direction to study the mechanism behind POAF.

## 5. Conclusions

In conclusion, the combined model, which includes clinical characteristics, radiomics signature, and features on non-enhanced CT images of EAT, demonstrated superior performance in predicting POAF risk after OPCABG. This suggests that the combination of these factors could be a valuable tool for improving POAF risk prediction.

## Data Availability

The raw data in this study can be obtained from the corresponding authors upon request.
